# High-Dose Glycine Treatment of Refractory Obsessive-Compulsive Disorder and Body Dysmorphic Disorder in a 5-Year Period

**DOI:** 10.1155/2009/768398

**Published:** 2010-02-18

**Authors:** W. Louis Cleveland, Robert L. DeLaPaz, Rashid A. Fawwaz, Roger S. Challop

**Affiliations:** ^1^Department of Medicine, St. Luke's-Roosevelt Hospital Center, Columbia University, New York, NY 10019, USA; ^2^Department of Radiology, New York-Presbyterian Hospital, Columbia-Presbyterian Medical Center, New York, NY 10032, USA; ^3^Department of Pediatrics, New York-Presbyterian Hospital, Columbia-Presbyterian Medical Center, New York, NY 10032, USA

## Abstract

This paper describes an individual who was diagnosed with obsessive-compulsive disorder (OCD) and body dysmorphic disorder (BDD) at age 17 when education was discontinued. By age 19, he was housebound without social contacts except for parents. Adequate trials of three selective serotonin reuptake inhibitors, two with atypical neuroleptics, were ineffective. Major exacerbations following ear infections involving Group A *β*-hemolytic streptococcus at ages 19 and 20 led to intravenous immune globulin therapy, which was also ineffective. At age 22, another severe exacerbation followed antibiotic treatment for *H. pylori.* This led to a hypothesis that postulates deficient signal transduction by the N-methyl-D-aspartate receptor (NMDAR). Treatment with glycine, an NMDAR coagonist, over 5 years led to robust reduction of OCD/BDD signs and symptoms except for partial relapses during treatment cessation. Education and social life were resumed and evidence suggests improved cognition. Our findings motivate further study of glycine treatment of OCD and BDD.

## 1. Introduction

Obsessive Compulsive Disorder (OCD) is an illness that is characterized by recurrent obsessions and compulsions that cause marked distress and impairment [1]. It has a lifetime prevalence of 2-3% and is the fourth most prevalent psychiatric disorder [2]. Estimates suggest that it is the tenth leading cause of disability in the world [3]. Up to 30–40% of patients fail to respond significantly to current treatments and those who do respond often experience only partial remissions [4, 5]. Patients who fail to respond to two serotonin reuptake inhibitors (SSRIs) are considered “refractory” [6]. Refractory OCD is regarded as an especially refractory disorder [7, 8] with severe cases being among the few psychiatric disorders that continue to be treated by irreversible procedures such surgical interventions, gamma knife ablation, and deep-brain stimulation with permanently implanted electrodes [9–11]. Given the nature of these treatments, there is an urgent need for effective pharmacotherapy. 

 The etiology of OCD is unknown. However, studies of clomipramine and desipramine revealed efficacy only for clomipramine [12, 13]. Since clomipramine has SSRI activity [13], it has been suggested that abnormalities of the serotonin system play a causal role in OCD. Although SSRIs remain first-line treatment for OCD and attempts have been made to associate polymorphisms in serotonin-related genes with OCD, no specific abnormality of the serotonin system has been definitively identified as a cause of OCD. The role of the dopamine system has also been considered and SSRIs have been augmented with neuroleptics, including atypical neuroleptics [4]. However, no specific abnormality of the dopamine system in OCD has been firmly identified. Recently, abnormal glutamatergic neurotransmission has also been considered as a basis for OCD. 

 In a theoretical analysis, Carlsson concluded that OCD and attention deficit hyperactivity disorder (ADHD) are antithetical with regard to phenomenology and with regard to prefrontal glutamatergic neurotransmission, with OCD being “hyperglutamatergic” and ADHD being “hypoglutamatergic” [14]. An experimental study using single-voxel proton magnetic resonance spectroscopy found that the combined glutamate/glutamine peak (GLX) in the anterior cingulate cortex is reduced in OCD [15]. Another study suggested that sequence variations in the 3′-untranslated region of the gene for the NMDAR-2B subunit (*GRIN2B*) are associated with OCD [16]. More recently, polymorphisms in the gene for the glutamate transporter (*SLC1A1*) have been associated with OCD in two studies [17, 18]. Taken together, these findings raise interest in abnormal glutamate neurotransmission as an aspect of OCD, but much work needs to be done to clarify the reproducibility of the associations and the functional significance of the polymorphisms. 

 Therapeutic approaches to OCD have also been attempted with reagents that modify glutamate neurotransmission. For example, riluzole [19] was used to augment pre-existing medication in an open 12-week trial with 12 treatment-resistant subjects. N-acetylcysteine [20] has been used with reported success to augment an SSRI in a case of refractory OCD. Topiramate augmentation of paroxetine has been reported to be beneficial in one case of refractory OCD [21]; whereas it appeared to cause OCD in two other cases [22, 23]. All of the foregoing trials are confounded by the use of more than one psychotropic reagent. To our knowledge, there are no reports of modulators of glutamate neurotransmission being used alone in a sustained fashion to treat OCD. 

 In contrast to approaches based on neurotransmitters, Swedo and coworkers have proposed that a subtype of OCD is the result of an antigen-specific autoimmune response that is stimulated by GABHS infection [24–26]. This diagnostic subtype is referred to as (pediatric autoimmune disorders associated with strep) PANDAS and may include OCD or Tourette syndrome as well as other comorbidities. Predictions of the PANDAS hypothesis for therapy have been tested in trials with IVIG and plasma exchange. Positive findings were initially reported [25, 26]. However, in the nine years following the placebo-controlled trial [26], these treatments do not appear to have become widely used, raising questions about the generality of the initial findings. 

 Body Dysmorphic Disorder (BDD) is an illness of unknown etiology that is characterized by excessive preoccupation with an imagined or minor defect in body appearance [27]. The preoccupation creates substantial distress or impairment. BDD is often a severe disorder and has been associated with an elevated suicide rate [28]. In a nationwide study in Germany, BDD was found to have a current prevalence of 1.7% [28]. BDD is a poorly studied disorder for which available treatments are frequently inadequate, with 27–47% failing to respond to adequate trials of SSRIs [29]. It is of interest that antipsychotics, either as monotherapy or as SSRI augmenters, are reported to be ineffective for BDD [29]. BDD is not unusual as a comorbidity of OCD, occurring in 12% of cases [30]. The combination of OCD and BDD is usually found to exhibit more impairment and a poorer prognosis than OCD alone [30–32]. 

 In this paper, we describe a case of refractory OCD and BDD in which infection-triggered exacerbations appear to have played a prominent role. A serendipitous observation of an exacerbation that followed treatment with a commonly used antibiotic has led to an hypothesis that postulates a deficiency in the NMDAR signal transduction cascade. This hypothesis points to a new direction of research on the molecular basis of OCD and BDD and makes the clear prediction that enhancement of NMDAR neurotransmission should provide amelioration of symptoms. In this paper, we present the observations from a five-year period in which glycine, an NMDAR coagonist, was used as the sole treatment of previously refractory OCD and BDD.

## 2. Methods

This study has been conducted under a protocol approved by the Institutional Review Board of the Institute for Health Sciences of St. Luke's-Roosevelt Hospital Center. Written informed consent under the aegis of this protocol has been obtained for the publication of the historical information in this paper. In addition, the subject (referred to as “O” in what follows) has read this paper and has given additional written informed consent for the publication of his case details. To protect subject identity, precise values of clinical tests with numerical results, which could be used as identifiers, have been replaced with approximate values indicated by “~”. Differences between modified and actual values are sufficiently small to be of no scientific significance. 

 A key resource for this naturalistic study has been a family-compiled archive that includes behavioral and medical information from birth to the present time. This information includes extensive written and recorded oral diary entries by O's father as well as written entries by O. In addition to blood test results, there are either reports or copies of charts from specialists and copies of charts of primary care physicians (from birth to the present). Photocopies of some prescriptions and pharmacy records covering the period when psychotropic medications were used are also available. In addition, tablet containers for psychotropic medications and some other medications have been retained as a primary record of medication prescribed. Detailed records of compliance monitoring are also available. Academic reports from kindergarten to the present, many examples of school work done by O, records of formal cognitive testing at ages 7 and 30, and results on standardized, US academic tests are also available. A valuable feature of O's archive is the existence of redundant information on many issues of critical importance. This has made it possible to identify and resolve discrepancies. 

 An important category of information in the archive is objective information from individuals or organizations that are unconnected with this study (referred to in what follows as “third-party” objective information). This category is free of concerns about bias arising from the fact that the subject, the subject's parents, and the investigators were not blinded in relation to glycine treatment. This category of information has been essential to establish the claim that there was major and persistent impairment before glycine treatment and substantial reduction of impairment following treatment. It has also been essential for establishing the occurrence of a substantial relapse in a period of nontreatment. 

 The archive also contains recorded observations of objective phenomena made by the subject or his parents. O's father reports that he has given emphasis to objective and qualitative indications of changes in behavior that were sustained over time. This information has been evaluated for its internal consistency and its consistency with third-party objective information. Subjective information (e.g., O's symptom reports and parental judgments) has also been evaluated for internal consistency and consistency with third-party objective information. 

 In cases where observations were recorded or recalled from memory at substantial time intervals after events occurred, this is explicitly indicated in the text. Moreover, serial interviews have been used to check the consistency of such recollections. A major effort has also been made to determine when observations could represent concept-driven perceptions or represent concept-driven selection of data. Examples of relevance to the conclusions of this paper are discussed in the text. 

 General medical history, radiological findings, and descriptions of OCD- and BDD-related behavior are included in an appendix, since this information may be useful for future definitions of glycine-responsive subtypes of OCD and BDD and as constraints on molecular models. Additional documentary material on methods and behavioral phenomena is at www.informaticpsychiatry.org/glycine.

## 3. Results

### 3.1. Time Course of Psychiatric Illness from Early Childhood to Glycine Treatment

O's parents recall their child as happy and contented for the first two years of his life. However, at age 2, they recall the sudden eruption of separation anxiety that was much more intense than what they saw in other children in their social group. The prodrome of O's illness became more clearly apparent at age 7 when he received psychotherapy from a psychiatrist whose chart noted “anxiety enormous.” At this time, learning difficulties in school prompted a request for cognitive testing, which revealed evidence for mild cognitive deficits. With improved school performance and social adjustment, psychotherapy was discontinued after one year. 

 As an adult, after developing an understanding of his illness, O has recalled wall-touching rituals as early as age 9, but these were not noticed by parents. O's parents believe they can recall several very mild examples of obsessive mentation that involved reassurance rituals as early as age 9. At age 14, they clearly recall a wall-touching ritual. Although they report that they were unaware of the concept of obsessive-compulsive disorder at that time, they regarded this behavior as abnormal and initiated a psychiatric consult. However, parental records and the psychiatrist's chart indicate that a diagnosis of OCD was not obtained at this time. 

 The mild prodrome in the prepubertal years was followed by frank illness that emerged rather abruptly at age 15 when school attendance was disrupted. After attending ~1/2 of ninth grade, O and his parents report that he refused to attend school any longer after developing the fear that students in the school had weapons. However, the presence of weapons was denied by school officials. This fear of crime was later found to be associated with a powerful line-crossing obsession that involved the fear that individuals likely to commit crimes were entering into his neighborhood. This obsession was associated with reassurance rituals referred to as “talk-throughs” (Appendix A.1.1). At this time, cognitive behavior therapy was attempted by a psychologist who is reported by O's parents to have found a mirror phobia and mild anxiety. No diagnosis of OCD or BDD was given. Attempts by the psychologist to treat the putative mirror phobia with a desensitization protocol are reported to have failed, and both O and his parents currently consider that “working on the mirror” intensified rather than reduced the mentation and behavior that was later diagnosed as BDD. With the endorsement of the psychologist, O was sent to boarding school where fears of ugliness and the “mirror phobia” are reported by both O and his parents to have intensified. When O returned home on vacation, he refused to return to school because of the “mirror problem.” Additional efforts by the psychologist to achieve mirror desensitization were again unsuccessful and led to psychiatric consultations that resulted in diagnoses of OCD and BDD at age 17. 

 Manifestations of BDD before age 15 are not recalled by O's parents. O currently recalls being mildly disturbed by his seventh grade class picture (age 13). In any case, available evidence suggests that BDD was preceded by obsessive-compulsive behaviors, which were preceded by cognition deficits. 

 In response to the diagnoses of OCD and BDD, SSRI therapy was prescribed. A trial of fluoxetine (40–80 mg/day, 134 days) was ineffective and was followed by a long trial of fluvoxamine (200–300 mg/day, 251 days). A significant response with the latter was also not obtained. A second opinion from a research psychiatrist specializing in anxiety disorders confirmed the diagnoses of OCD and BDD and recommended risperidone as an adjunct to fluvoxamine. 

 At age 19, during the trial of fluvoxamine (300 mg/day) + risperidone (2 mg/day), a severe exacerbation occurred that involved intensification of pre-existing symptoms and the emergence of terrifying, violent imagery, a symptom which had not been reported before. After full completion of the recommended 30-day risperidone trial, diary records indicate a decision by O's parents to suspend pharmacotherapy on the basis of their concern that this exacerbation was a reaction to the medication. Psychotherapy was subsequently initiated with a different psychiatrist and was continued for about nine months. Following the age-19 exacerbation, O is reported to have become largely housebound as a result of the line-crossing obsession associated with a fear of crime (Appendix A.1.1). He left his home rarely and only with parental escort. The addition of BDD by proxy to pre-existing self-related BDD also occurred at this time. BDD by proxy involved O's fear/belief that his parents were ugly and that he looked like them (Appendix A.1.2). Also present were somatic/cognitive preoccupations that were very mild in relation to other symptoms (Appendices A.1.3 and A.1.4). One year after the age-19 exacerbation, there was another exacerbation similar to the previous one in its severity and in the emergence of terrifying violent imagery. However, in this exacerbation, no psychotropic medication had been taken in the past nine months. 

 After further deterioration following the age-20 exacerbation, another OCD expert was consulted. The diagnoses of OCD and BDD were confirmed. Paroxetine (50–70 mg/day) and olanzapine (5 mg/day) were prescribed and O was referred to another psychiatrist for behavior therapy and weekly monitoring. The latter soon added clonazepam (0.5–4.0 mg/day). In spite of an initial, apparent improvement that permitted O to go for short, unescorted walks in his neighborhood during a three-week period, a clear deterioration was apparent after 3.5 months, leading the psychiatrist to terminate olanzapine and to continue paroxetine at 60–70 mg/day for another 9 months. Again no improvement was perceived. 

 In response to the failure to make progress with established pharmacotherapy, O and his family began to search for newly emerging treatments. When O was 21, they became aware of the PANDAS concept of Swedo and coworkers [24–26]. Consultation with O's pediatrician revealed that two severe cases of bilateral ear infections with *β*-hemolytic-positive throat cultures had occurred approximately 1-2 months before the peaks of the age-19 and age-20 exacerbations (*β*-hemolytic-positive throat cultures are ~50% probable GABHS infection in temperate climates, J.B. Zabriskie, personal communication). O's father's diary contains a detailed description of the age-19 exacerbation, which began as soon as the infection cleared. Seeing their son's further deterioration, O's parents agreed to augmentation of fluvoxamine with an atypical neuroleptic. O's father emphasizes that at that time he had no knowledge of the PANDAS concept and therefore did not associate the deterioration with the *β*-hemolytic-positive infection that was discovered retrospectively two years later. 

 The retrospective discovery at age 21 that probable GABHS infections preceded the age-19 and age-20 exacerbations led to consultations with a rheumatologist. In response to a report that IVIG treatment was effective with PANDAS [26], IVIG therapy was initiated. Paroxetine was tapered from 60 mg/day to zero and overlapped IVIG for only two months. IVIG therapy was initiated at 160 grams for the first month and 80 grams/month for an additional 17 months. Although there was an apparent behavioral improvement after the first infusion, this improvement was not sustained and IVIG was eventually terminated for lack of efficacy. About 3 months after the IVIG trial began, 500 mg/day of amoxicillin was added as a prophylaxis against GABHS. O's parents report no obvious benefit, and also no obvious deterioration, from amoxicillin prophylaxis, which was terminated after about 6 months to permit initiation of a different antibiotic regimen to remove *H. pylori*. 

 Nine months into the IVIG treatment, removal of *H. pylori* using a combination of clarithromycin (1000 mg/day), amoxicillin (1500 mg/day), and lansoprazole (30 mg/day) for 12 days was associated with a severe exacerbation of existing obsessions and compulsions and emergence of highly distressful violent imagery. Episodes of highly disturbing violent imagery are reported to be extremely distinctive, occurring only three times in O's life, namely in this exacerbation at age 22 and in the two major exacerbations at ages 19 and 20 that followed severe bilateral ear infections. Another distinctive feature of this exacerbation was a much stronger presentation of ritualistic behaviors than ever seen before, either during baseline illness or during previous exacerbations. O's father's diary indicates that the main ritual was a prayer ritual that was performed for many hours a day, leading initially to knee erosions that were followed by calluses. Another distinctive feature of this exacerbation is its similarity to the previous ones in which there was a gradual rise in symptoms over a period of approximately 1-2 months. Before, during, and after the antibiotic treatment, measurements of streptolysin O titers were negative. After the peak of the exacerbation, records indicate that the prayer ritual gradually declined over a period of approximately 12 months to generate a new baseline in which ritualistic behaviors are reported to have been increased relative to pre-exacerbation levels. 

 After the termination of IVIG treatment, O's parents report that they decided to support their son at home on their own until a new treatment was developed. While waiting for the development of new treatments (from age 23 to 25), O and his parents tried on their own initiative and in consultation with physicians various nutraceutical treatments, (e.g., [33, 34]) that had been developed for other illnesses that are sometimes associated with OCD, such as Huntington's disease [35]. However, none showed efficacy. The glycine trial described below represented another of their efforts involving the use of a nutraceutical.

### 3.2. Stability of Psychiatric Diagnoses

The diagnoses of OCD and BDD that were given at age 17 by a psychiatrist were subsequently confirmed by several other psychiatrists, including two OCD specialists. No signs or symptoms of other major psychiatric illnesses have been noted by the multiple psychiatrists who have examined O. There is no evidence of substance misuse, as noted in *General Medical History* (Appendix A.2). In O's continuous and generally increasing illness, individual signs and symptoms have varied in intensity over time. However, the basic spectrum of signs and symptoms appears to have been unchanged since the emergence of frank illness at age 15, suggesting stable diagnoses from age 17 to age 30.

### 3.3. Development of Hypo-NMDAR Signal Transduction Hypothesis

At the time of the exacerbation that followed *H. pylori* eradication, it was considered that the exacerbation could have been due to one or more of the three agents used to achieve eradication (amoxicillin, lanzoprazole, clarithromycin). Clear precedents for psychiatric sequelae of lanzoprazole and amoxicillin were not found in the literature at that time. More recent literature suggests that beta-lactams can modulate glutamate neurotransmission but that doses substantially above the therapeutic range appear to be required [36]. Consistent with this finding is the observation that O has taken amoxicillin on multiple occasions before and after the exacerbation without adverse effects. In contrast to amoxicillin and lanzoprazole, there were reports describing substantial CNS and psychiatric sequelae from macrolide antibiotics, including clarithromycin. However, a majority of reports on psychiatric sequelae from clarithromycin claimed remission immediately after cessation of treatment. In O's case, the exacerbation continued to increase over a period of approximately 2 months after the 12-day treatment period. Consequently, the role of clarithromycin was discounted until a reanalysis three years later in 2002. 

 In 2002, one of us (W. L. Cleveland) became aware of studies of phencyclidine's effects on patients with medication-controlled schizophrenia. A remarkable finding is that a subpopulation of these patients can exhibit a prolonged exacerbation following a single, brief exposure to the drug. This observation was specific for phencyclidine, an NMDAR inhibitor. Drugs interacting with other receptors, such as LSD, failed to exhibit protracted exacerbations (see [37, 38] and references therein). The similarity of the protracted kinetics of the phencyclidine response to the kinetics of the antibiotic-induced exacerbation seen in O led to a literature search for evidence that clarithromycin could influence NMDAR neurotransmission. A paper by Manev et al. provided evidence that clarithromycin could inhibit cell death from glutamate-induced excitotoxic injury in cultures of human cerebellar neurons [39]. Protection was against NMDA-mediated but not kainate-mediated excitoxicity. Activation of the NMDAR as indicated by calcium influx was not inhibited, suggesting that clarithromycin (and the other macrolides studied) inhibited a downstream aspect of the NMDAR signal transduction (NMDAR-ST) cascade. 

 The physiological relevance of the data of Manev et al. was also analyzed. The concentrations used in their in vitro studies were in the range of 100 *μ*M. The literature indicates that clarithromycin can reach comparably high concentrations in tissues and cells, with concentration increasing in proportion to the duration of treatment [40]. Moreover, access of clarithromycin and its main, active metabolite, 14-OH-clarithromycin, to the brain is suggested by the observation that clarithromycin is useful in the treatment of central nervous system toxoplasmosis infections [41, 42]. It should be noted that low concentrations of macrolides in cerebrospinal fluid (CSF) are an indication of a marked tendency for macrolides to accumulate in cells rather than poor access to the brain. For example, a study of azithromycin found ~3 *μ*g/g in brain tissue, whereas in CSF it was barely detectable [43]. The macrolide affinity for cells is also seen in a comparison of clarithromycin and azithromycin, which reached levels of 940 *μ*M and 520 *μ*M, respectively, in alveolar macrophages [40]. 

 The above considerations led to the hypothesis that clarithromycin induced an exacerbation by inhibition of a downstream aspect of the NMDAR-ST cascade. Moreover, it seemed plausible that this inhibition modulated, either directly or indirectly, the intrinsic defect responsible for baseline symptoms. An immediate prediction of this hypothesis is that an NMDAR agonist (or coagonist) would lead to symptom improvement. This prediction motivated consideration of the high-dose glycine treatment developed for schizophrenia, which has been used with apparent safety as an adjunct to other medication in trials beginning in 1988 [44, 45]. 

 After consultation with physicians, O decided to initiate a trial of glycine at 0.8 grams/kilogram of body weight/day, the dose used with apparent safety in schizophrenia trials [45]. The initial response to glycine was monitored in relation to major life disruptions (see below) and in relation to the specific signs and symptoms of preglycine illness (Appendix A.1).

### 3.4. *Major Life Disruptions*

**School**

A sign of major impairment is no attendance of high school after failing to complete tenth grade. An attempt to continue O's education with one-on-one tutoring was discontinued six months after stopping regular school. O, however, did make sporadic attempts to study at home. This led to an unsuccessful attempt to take GED examination (for high school equivalency diploma) at age 25, several months before initiation of glycine treatment. Records indicate that when O arrived at the test site, he refused to enter, giving an explanation related to his line-crossing obsession. At this time, he also refused to handle photo-identification for entry into the test site. Thus, in the 8-year period preceding glycine treatment (age 17 to age 25), there was no regular school or formal tutoring.

**Social Life**

The diary maintained by O's father indicates that by age 19, O had become housebound except for doctor visits, visits to his father's place of work, and rare visits to an art museum, all of which required his father's escort and door-to-door taxi travel along routes that were perseveratively selected by O because of the fear that his line-crossing obsession would be triggered by people he might see on alternate routes. At age 21, there was a brief period of about 3 weeks duration, during treatment with paroxetine + olanzapine, in which O went for short, unaccompanied walks in his neighborhood. Otherwise, records indicate that O was housebound in the six-year preglycine period that followed the age-19 exacerbation. His only in-person social contacts were with his parents at home. There were no other social contacts except for telephone contact with a grandmother in another city.

**Behavior at Home**

O's disorganized lifestyle is reported to have created disarray in his home that prevented O's parents from bringing guests to their home for at least seven years preceding glycine treatment. There was also poor personal hygiene and grooming. Most of O's attention was on television and the internet. His sleep cycle was frequently inverted. O's made some effort to do school work, but he was generally resistive to parental tutoring. Any attempt to discuss academics was thwarted by the tendency to discuss an obsession.

The last exacerbation in the preglycine period occurred 3 years before initiation of glycine treatment. As noted previously, the decline of this exacerbation over about one year led to a baseline in which ritualistic behaviors were increased relative to the pre-exacerbation baseline. This new baseline remained stable in the two years preceding the initiation of glycine treatment. It was against this stable baseline of clear and substantial impairment involving the above life disruptions that the response to glycine treatment was initially detected.

### 3.5. Response to Glycine Therapy

In this 5-year study of glycine treatment, decisions to start and stop glycine treatment were made by O in consultation with physicians. Table 1 lists observation periods and the corresponding glycine doses. Target doses varied from 50 to 66 g/day (0.6–0.8 grams of glycine per kilogram of body weight). As an indication of compliance, the percentages of total days for various dose ranges are given. Average daily doses for observation periods are also given. Tables 2 and 3 summarize behavioral and cognitive changes, respectively.

#### 3.5.1. Observation Period 1 (on Glycine for 203 Days, 1–203)

Glycine treatment was typically initiated by increasing the dose over 12 days from 10 g per day to the target dose. In Observation Period 1 (OP1), the full dose was initially taken t.i.d., but, for reasons of convenience, was subsequently taken b.i.d. The daily target dose was varied from 50 to 66 g/day in this period. Glycine was abruptly terminated on day 204 due to an upper respiratory infection with high fever. 

 O's father's diary indicates that the first improvement was seen 23 days after reaching target dose on Day 34-OP1(34) (Day 34 of Observation Period 1(Day 34 of Study)). For the first time in 5 years, O left his apartment building without parental escort and went for a short walk in front of his building. Soon after this, he began daily attendance a nearby library where he prepared for the high school equivalency exam (General Education Development (GED) test of the American Council of Education). This is reported to have been done on a 9 AM to 5 PM weekday schedule without parental escort. 

 The GED exam was passed on Day 145-OP1(145) in the standard time period. Official scores on the five GED subtests ranged from the 67th to the 99th percentile on scales normalized to scores of graduating high school seniors in the US. 

 Reduced intolerance to mirrors is supported by a sustained resumption of barbershop haircuts after approximately five months of glycine treatment. O was escorted to barbershops by his father, who reports that he observed no obvious distress over mirrors. Haircuts had been given at home for the past seven years. 

 Six months after initiation of glycine treatment, O was encouraged to study in a different library on the campus of a university near his home. Reaching the campus required paternal escort and crossing a busy street that had previously been avoided due to the line-crossing obsession. Entry required the presentation of a photo-identification card. This was an improvement over the preglycine period in which O insisted that his father handle his photo-identification. 

 O's parents consider that the partial tolerance of the street and campus indicated a significant and further reduction of the line-crossing obsession. At this time, O's father's diary indicates that talk-throughs, which had been daily before glycine treatment, had become infrequent. The willingness to handle a photo-identification card is taken as a sign of a reduction of BDD. 

 Although substantial illness remained, the data for OP1 suggest steady and significant progress during the first 200 days of glycine treatment.

#### 3.5.2. *Observation Period 2 (off Glycine for 41 Days, 204–244*)

On Day 1-OP2(204), O developed an upper respiratory infection with fever as high as 104.2°F (oral, Hg thermometer). A commercial lateral flow immunoassay for GABHS antigens (One-Step ClearVue, Quidel, San Diego, CA) was negative. However, a course of augmentin was prescribed. Since this fever was thought to be unusually high in relation to O's prior history, O and his parents became concerned that it could be due to glycine. This led to the discontinuation of glycine treatment for 40 days. 

 Detailed notes in O's father's diary suggest a gradually rising exacerbation following the infection. On Day 21-OP2(224), the line-crossing obsession was activated on the way home from the library, leading to the first talk-through in quite sometime. The next day, it took an hour of parental urging to get O to leave his home. O reported the emergence of a hand-washing ritual driven by fear of harm. Talk-throughs again became a daily occurrence. O's parents describe an unequivocal but not major loss of prior gains. Although unequivocal, they described this exacerbation as distinctly less intense than the major exacerbations at ages 19, 20, and 22. Most notably, it did not involve terrifying violent imagery. 

 By the time of this exacerbation, both O and his parents were aware of the concept that exacerbations could result from infections. Therefore, there is the possibility that the exacerbation described for this period reflects concept-driven perceptions or selection of data. However, O's father notes that, at that time, they expected exacerbations only from GABHS infections, since the two previous infection-associated exacerbations were associated with positive throat cultures. Therefore, as a result of the negative test for GABHS done at the beginning of the infection in the office of O's primary care physician, an exacerbation is described as unexpected.

#### 3.5.3. Observation Period 3 (on Glycine for 127 Days, 245–371)

In response to the deterioration seen in OP2, O and his family decided to resume glycine treatment on Day 1-OP3(245). After 5 days at 25 g/day, glycine was again terminated because of another infection with chills and vomiting and a fever of 102.4°F. No antibiotic was taken. Glycine was again resumed at full dose (50 g/day) on Day 13-OP3(257). On Day 21-OP3(265), O reported a reduction in rituals. Fewer rituals and fewer complaints related to the line-crossing obsession were noted on Day 29-OP3(273). Other observations suggest a rapid recovery of lost gains. On Day 31-OP3(275), O was escorted only to the edge of the campus, walking to the library by himself. The trend to improvement is further suggested by the fact that by Day 85-OP3(329), O began freely exploring the entire campus. On Day 91-OP3(335), he began working with a tutor (other than his parents) for the first time in 9 years. Records indicate that talk-throughs again became rare. Increased tolerance of his mother's face is suggested by attendance with her at movies and restaurants and acceptance of her help with SAT preparation. 

 Third-party records indicate that a major activity during this period was a preparatory course for the SAT, a reasoning test of The College Board required for admission by many US colleges. Course attendance involved classroom participation and practice tests under conditions simulating official test conditions. O's tolerance of a classroom with many students suggests a reduction of BDD-related social discomfort. Travel to the course site was by taxi with parental escort, often O's mother. Moreover, the course site was on a street that was avoided in the preglycine period due to the line-crossing obsession. 

 Third-party course records indicate that O's math scores in the practice tests increased by 100 points and his verbal scores by 60 points. According to The College Board, a difference of 60 points is evidence of a true difference in ability (“standard error of the difference”) [46]. 

 In this period, the archive lists a dozen “landmark” events in which O resumed an activity that had been prevented in the preglycine period. Even though they acknowledge that substantial illness remained, O's parents report that they considered the progress achieved by the end of this observation period to be a dramatic improvement over preglycine illness.

#### 3.5.4. Observation Period 4 (off Glycine for 40 Days, 372–411)

Given that the exacerbation of OP2 was associated with both an infection with high fever and a cessation of glycine treatment, it was unclear if glycine cessation contributed to the exacerbation. Therefore, O and his family decided to stop glycine treatment during a period without infections. No significant change was observed in this period.

#### 3.5.5. Observation Period 5 (on Glycine for 138 Days, 412–548)

In this period O made his final efforts to prepare for the SAT that was taken on Day 68-OP5(479). Scores on the actual tests, which were taken in the standard time intervals, surpassed all practice test scores. His verbal score was in the 90th percentile and was 60 points higher than the best practice test and 120 points higher than the first practice test. As noted above, a 60-point increase is statistically meaningful. His math score was at the 50th percentile and was 30 points better than the best practice score and 130 points higher than the lowest practice test. 

 O's improved life style at home led his parents to renovate their home and resume normal family social life for the first time in 8 years. Diary records indicate that he was present for the first visit of a family friend to his home in five years. 

 In this observation period, O began to walk freely in his neighborhood without any escort. This step forward is among the 10 “landmark” occasions in the archive where O resumed an activity prevented in preglycine period. Talk-throughs largely disappeared in this period. Barbershop haircuts continued. 

 OP5 is important because it reveals the robustness and stress-resistance of O's improvements. On Day 68-OP5(479) there was the SAT. This was followed by application to college which required an interview with an admissions official on Day 112-OP5(523). Although the interview is reported to have gone well, O's application was rejected on Day 123-OP5(534). On Day 137-OP5(548) O attended an alternative college that was located at a site that would have been a major line-crossing trigger in the preglycine period. In response to this sequence of major stressors, O's father reports that he saw no loss of gains. Taken together, the data for OP5 suggest further substantial improvements that were a continuation of gains seen in OP3.

#### 3.5.6. Observation Period 6 (off Glycine for 220 Days, 549–768)

As described in OP2, an upper respiratory infection with high fever was followed by a behavioral exacerbation. A concern that glycine might have caused the unusually high fever led to the decision to stop glycine during the flu season. The extension beyond the flu season was due to O's reluctance, arising from the unknown safety of long-term, high-dose glycine therapy. 

 It was at the beginning of this observation period that O resumed his formal education after not attending school for an entire decade. As noted above, O was not accepted in the college of his choice. However, the letter from the Admissions Office encouraged reapplication after demonstrating competence at another institution. O thus felt under pressure to obtain high grades. Two courses, College Algebra and English, were taken. The archive indicates that O attended all classes, completed all assignments on time, and received no special accommodations. He is reported to have tolerated the stress associated with academic deadlines, including all-night sessions to complete papers. Academic records document very high grades that permitted a successful reapplication to the college of his first choice. 

 In this 4.5-month period of glycine cessation, there were two indications of a loss of prior gains: a decline in cognition (see Section 3.5.11) and a mild increase in the line-crossing obsession. There was also an intensification of somatic/cognitive preoccupations to the point that they generated (for the first time) mild impairment. A notable feature of OP6, the first lengthy (220-day) off-glycine period is that the observed, mild relapse in OCD was circumscribed and occurred slowly.

#### 3.5.7. Observation Period 7 (on Glycine for 254 Days, 769–1022)

The decision to resume glycine is reported to have been due to multiple considerations. In addition to a general perception of relapse by O's parents, there was also a major concern about the impact of the apparent cognitive decline seen in OP6 (see Section 3.5.11) on academic performance in a new college with a more demanding curriculum. While O's parents report that they strongly encouraged resumption of glycine therapy, the final decision was left to O as in the past. 

 Attendance at the new college began on Day 13-OP7(781) with O taking two courses. One course proved to be quite difficult for O. A low grade on the first exam of this course was obtained on Day 40-OP7(808). This is reported to have generated considerable stress, since O as a new student with an unusual academic and personal history was under pressure to demonstrate his ability to handle the curriculum. O's father's diary indicates that there were no signs of a behavioral relapse from this stressful experience. O finished the semester with a respectable grade in the other course. He is reported to have handed in all assignments on time and to have attended all classes except for one. Stresses from academic deadlines, for example, all-night sessions to complete papers, are reported to have been handled without any sign of a behavioral deterioration. In the second semester, academic records show that the same consistently adequate performance continued with respectable grades. 

 An important BDD-related gain in this period is O's reported resumption of hair combing in front of a mirror. Although O covered his nose and mouth with one hand, this maneuver suggests an increased tolerance of mirrors, since, prior to this period, hair combing was reportedly done with parental assistance or by manual feeling without visual feedback. O's father reports that this maneuver did not involve any repetitive “working on the mirror,” as hair combing was done quickly. Barbershop haircuts continued. 

 Another BDD-related “landmark” event in this observation period is seen in an entry in O's father's diary on Day 34-OP7(802), which indicates that O began to tolerate direct observation of his father's face in a sustained manner. Prior to this, tolerance of direct observation is reported by O to have occurred but only sporadically. O tolerated his mother's face during multiple social events in this period. However, he continued to maintain that he kept her face in peripheral vision. 

 The line-crossing obsession appears to have become much reduced in this period. O's father's diary notes only a single talk-through dealing with this obsession. The only obvious remnant of the line-crossing obsession was a partial restriction of the use of public transportation. 

 In summary, there were notable gains that involved coping effectively with a new and challenging academic curriculum, a further reduction in the line-crossing obsession and reductions in self-related BDD and BDD by proxy. Somatic/cognitive preoccupations appear to have remained mild without any clear change. Importantly, multiple stressful events did not lead to any obvious loss of behavioral improvement.

#### 3.5.8. Observation Period 8 (off Glycine for 46 Days, 1023–1068)

The decision to stop glycine was as always made by O. The reasons were not recorded. O's father reports that he was not able to detect any consistent trend toward deterioration or toward improvement.

#### 3.5.9. Observation Period 9 (on Glycine for 331 Days, Days 1069–1400)

Eighty three days after glycine resumption (Day 83-OP9(1151), O began the third semester at his new college, registering for two courses. Academic records indicate that O obtained the highest possible grade in one course and next to the highest possible grade in the other course. No courses were dropped. On Day 216-OP9(1284), the fourth semester began. As before, two courses were taken and the same excellent grades were obtained. 

 Diary entries and email correspondence indicate that social activities occurred in classes, in student centers, at social events for students, and at athletic events. All of these activities, except for in-class socialization, are reported to have occurred for the first time in this semester. On Day 202-OP9(1270), O went on his first date with a girlfriend in 13 years. 

 Also of interest in OP9 are records indicating a substantial reduction in the signs of BDD by proxy involving O's mother's face and in the signs of BDD involving his own face. On Day 41-OP9(1109), O announced that he was able to look directly at a mirror image of his face with only his mouth and the bottom surface of his nose covered. Previously, he had found it necessary to cover his nose and lower part of his face. Another indication of reduced BDD intensity is the occurrence of very few BDD-related talk-throughs in OP9. O's father's diary lists 13 talk-throughs for the entire 47-week period. Only several of these were BDD-related. Taken together, the above observations suggest significant, additional reduction of BDD during OP9. 

 As noted above, the line-crossing obsession was considered by O and his parents to be substantially reduced by the end of OP7. In OP9, there are indications for the further reduction of this obsession. Most notably, O is reported to have begun freely traveling about his city using bus and subway transportation. This was done either alone or with peers without restrictions on time or areas traversed. O's father's diary records no talk-throughs regarding the line-crossing obsession in OP9. As in OP7, there were no signs of any sensitivity to crime news. Both O and his parents currently report the complete disappearance of the line-crossing obsession in OP9. 

 Somatic/cognitive preoccupations are reported in O's father's diary to have been the subject of most of the thirteen talk-throughs listed in this 331 day period. O's parents consider that somatic/cognitive preoccupations became milder in the final parts of OP9. This is supported by the absence of medical consults for somatic preoccupations in these periods. 

 On the basis of the excellent progress in this period, O and his parents report that glycine treatment would have been continued had it not been for the occurrence of unexplained weight loss. OP9 was terminated on Day 332-OP9(1400) to observe weight fluctuations in the absence of glycine therapy. 

 Taken together, the above observations for OP9 suggest the addition of major gains to those seen in OP7 and prior treatment periods. By the end of OP9, these gains represented a largely complete remission of OCD and a large fractional reduction in BDD-related impairment relative to preglycine illness. The reduced impairment reflected in the high level of O's academic and social activities represented a major reversal of the life disruptions of preglycine illness.

#### 3.5.10. Observation Period 10 (off Glycine for 541 Days, 1401–1941)

Although, unexplained weight loss initiated this period, O reports that concerns about the unknown safety of long-term treatment led him to extend it. 

 Available evidence for this long off-glycine period suggests the occurrence of a clear but mild relapse in BDD, a milder relapse in OCD, and a major relapse in cognition (see Section 3.5.11). 

 Clear signs of a relapse can be seen for self-related BDD. Specifically, O refused the opportunity to take driving lessons after the end of the academic semester (Day 372-OP10(1772)) on the basis that he might see his face in the mirrors or the windshield of the automobile. The opportunity to travel to a foreign country was refused on the grounds that he would not be able to handle his passport without seeing his picture, although he continued to handle old photo-identification. There was also a return of a hair-loss preoccupation seen in the preglycine period. O's parents consider the above signs to be a clear increase in BDD intensity and impairment that was modest compared to pretreatment illness. Additional diary entries suggest an increase in mother-related BDD by proxy that is reflected in a loss of O's ability to tolerate his mother's presence and the risk of seeing her face. 

 In OP10, there were no signs of a major return of the line-crossing obsession. However, two episodes suggest the beginning of a restriction in mobility. On Day 380-OP10(1780) O refused to meet his father in an area of a public park which he had previously visited without complaint. In a recent interview, O explained that he was afraid that he would see someone in that location who would trigger the return of the line-crossing obsession. On Day 362-OP10(1762) O refused to accompany his father to a restaurant in an area that had been highly sensitive in relation to the line-crossing obsession in the preglycine period. Diary records indicate that in OP9, O had gone to restaurants in the same area with a girlfriend. Some weeks later on Day 411-OP10(1811), a similar episode occurred. The above observations suggest a small restriction in mobility resulting from a mild return of the line-crossing obsession. 

 On Days 279-OP10(1679), 380-OP10(1780), 388-OP10(1788), O's father's diary contains descriptions of episodes which suggest a return of concerns about crime-related news. In a recent interview, O confirmed his father's observations. 

 Somatic/cognitive preoccupations in the first part of OP10 were not greatly changed from the previous observation period. However, complaints about speech difficulties increased in the last three months of this period, leading to a consult with a clinical neuropsychologist. The consult failed to identify speech problems but did identify memory deficits, which are discussed below in the section on cognition. 

 This observation period again revealed the slow nature of the off-glycine relapse that was seen in OP6, the previous lengthy off-glycine period. As before, the relapse of OCD was modest. BDD exhibited a more unequivocal relapse that clearly represented increased impairment, but the relapse was far from the preglycine baseline. Formal cognitive testing toward the end of this period revealed a much more dramatic relapse of the cognitive dimension that is likely to be close to preglycine baseline (see below).

#### 3.5.11. Effects of Glycine on Cognition

Thirteen days after ending a glycine treatment period of 138 days (OP5), O demonstrated rapid learning and retention of new math concepts in a tutoring session for a math placement exam given at the time of college enrollment. In this session with his father, his work was largely free of the types of errors seen by his father at 14 and also in formal cognitive testing at age 7. This parental impression was supported by higher than expected performance on the placement exam. During the ensuing algebra course, after an additional 21–26 weeks of glycine cessation, retention difficulties and the characteristic pattern of errors reappeared. These informal observations suggested the possibility of a cognitive benefit from glycine treatment that slowly declined after treatment was stopped. 

 The possibility of a cognitive benefit from glycine treatment was much more strongly suggested by third-party objective data derived from formal cognitive testing done at the end of OP10. Toward the end of OP10, O was evaluated by a neuropsychologist who was blinded to psychiatric and treatment histories during testing and the preparation of a preliminary report. 

 The evaluation indicated that fine motor skills and speed were within normal limits as were simple attention, mental manipulation, and sequencing. However, with more complex tasks requiring manipulation of nonsequential items, self-monitoring, response inhibition, and set shifting, performance declined considerably. Visual-perceptual and visuoconstructive skills were described as severely impaired. Visual memory was also severely defective, especially when learning material that lacked structure or associative quality. Verbal memory was described as “impaired” when dealing with material that was unstructured or unrelated. O's chief cognitive complaints concerning speech were not verified. On the contrary, oral expression received the highest score (94th percentile). 

 In this analysis of the clinical neuropsychological data, we focus on test results that are relevant to memory function. Of special interest are results from the Wechsler Memory Scale-III (WMS-III) [47] and the California Verbal Learning Test-II (CVLT-II) [48]. The WMS-III auditory immediate and auditory delayed subtest scores were in the ~1st percentile. General memory was at the “borderline” level. Memory deficits were also revealed by the CVLT-II, where all but two Z-scores ranged from ~−1 to ~−4. 

 In our view, these substantial off-glycine deficits are highly unlikely to have been present in the on-glycine periods when O obtained a verbal SAT score in the 90th percentile and a 99th percentile score in the reading and social studies subtests of the GED. Here we emphasize that both the GED and the SAT scores were obtained in the standard time periods. These test scores also seem to be in marked contrast with the psychologist's impression that attention and executive deficits at the time of testing (off-glycine for 17 months) exerted considerable impact on tasks requiring complex mental manipulation, self-monitoring, multitasking, and working memory. 

 Further support for a cognitive improvement from glycine treatment can be found in evidence that the off-glycine deficits seen in OP10 represent a decline from a prior time. Such evidence derives from the Wechsler Test of Adult Reading (WTAR) [49] in which word reading scores (thought to be resistant to brain injury or other causes of cognitive decline) are used to predict premorbid IQ scores. The WTAR presumes that adult reading levels were achieved before cognitive decline. Clear evidence for achievement of adult reading levels can be seen in O's 99th percentile score on the reading and social studies subtests of the GED (age 26) and his 90th percentile score on the verbal part of the SAT (age 27). 

 WTAR results (at age 30) obtained 17 months after glycine cessation indicate that O's actual Verbal standard score, Full-Scale standard score, Perceptual Organization Index score for the Wechsler Adult Intelligence Scale-III [50] were 14–17 points below WTAR predictions (cumulative percentage = 5–9%). A more dramatic difference of ~30 points between predicted and actual was seen for the Working Memory Index scores (cumulative percentage = 1%). Here it should be noted that working memory is central to cognitive functioning and is strongly correlated with reasoning ability [51]. Moreover, working memory is currently considered to be a key determiner of fluid intelligence [51, 52]. Given the central importance of working memory and O's high verbal score on the SAT (reasoning test of the College Board) at age 27 in OP5 (137-days on glycine) and the absence of any infection-triggered or other obvious exacerbations since that time, we suggest that the WTAR data are most consistent with a cognitive decline as a result of glycine cessation in the 17-month period that preceded the test. Table 3 summarizes educational and other cognitive data. 

 Another issue is the possible interference of obsessive mentation with test performance. O's father attended both testing sessions to address this issue. His recorded notes indicate that O reported no obsessions or mental rituals or any other discomforts or distractions in either testing session. O reported: “I gave it my all.” The clinical psychologist's report noted that O was euthymic and appeared to make a full effort and that test results for depression, anxiety, and ADHD indicated that these factors did not impact test results. Results were therefore judged to represent a valid picture of cognitive status. 

#### 3.5.12. Adverse Effects of High-Dose Glycine?

Ingested glycine appears to enter circulation rapidly, generating a sharp rise in plasma glycine concentration. In O's case, multiple measurements at approximately two hours after completion of consumption have yielded values ranging from ~2600 to ~3300 *μ*M, with an average of ~2800. The time at which peak concentration is reached is unknown, but may be less than 2 hours after ingestion and may depend on the rate at which glycine is consumed and on the presence of other food in the stomach. On one occasion, a plasma value obtained at 3 hours after glycine ingestion (~1350 *μ*M) was substantially less than values obtained at 2 hours (~2000–3000 *μ*M) (normal reference 151–490 *μ*M). On another occasion, 24 hours after terminating an extended period of glycine consumption, plasma glycine was ~280 *μ*M, a base-line value in the reference range (151–490 *μ*M). This observation is consistent with studies of healthy volunteers by Hahn and Sandfeldt, who found the half-life of excess plasma glycine to range from 40 to 100 minutes in many individuals to 6 hours in one individual [53]. 

 Hahn and Sandfeldt also found that glycine infusion in amounts comparable to those used in this study induced little or no increase of plasma ammonia in most individuals [54]. However, about 10–15% of healthy volunteers developed plasma ammonia levels in the 100 *μ*M range after i.v. infusion of 20 g of glycine [54]. O appears to be in this category. For example, in a typical measurement, plasma ammonia about 2 hours after glycine ingestion was ~120 *μ*M (reference: ≤47 *μ*M). Orotic acid in urine at this time was ~15 mmol/mol/creatinine (reference: 0.4–1.2). Several physicians, including specialists on hepatic encephalopathy and urea cycle disorders, have suggested that the mild hyperammonemia seen in O is unlikely to cause harm. Nonetheless, hyperammonemia represents an issue that, to our knowledge, has not been studied in prior trials. 

 As noted previously, OP1 was terminated because of a fever of 104.2°F during an upper respiratory infection. At that time, O and his parents could not recall a fever this high in previous infections and worried that glycine might cause elevated temperatures. However, no high fevers have been experienced in the subsequent four years, during which glycine was taken for 850 days. In this period, the only precaution has been to terminate glycine at the first sign of an infection. Although there is no clear evidence for glycine inducing higher-than-normal fevers, we include these findings for future reference, since there is literature suggesting a role of NMDAR-ST in the fever response to bacterial toxins [55]. 

 Mild weight loss with no obvious cause led to the termination of OP9. On the basis of sporadic measurements, weight declined from 184 pounds to 165 pounds over a period of 22 weeks. Glycine consumption was completely stopped and weight remained at this level for 17 weeks. Weight then increased to 172 pounds over a period of 8 weeks. Over the next 6 months, weight gradually returned to prior levels with no deliberate effort to increase weight. As noted in the section for OP10, there were no signs of anorexia nervosa or other eating disorders. At this point there is no clear evidence for glycine as the cause of weight loss. 

In summary, available evidence suggests that high-dose glycine treatment used for a total of 947 days in a five-year observation period has not produced any detectable, serious adverse affects in an individual closely monitored by his physicians. This observation is in agreement with the apparent absence of serious adverse effects in the 20 years of glycine trials with schizophrenia [56].

## 4. Discussion

### 4.1. The Results of This Case Study Are Consistent with the Possible Efficacy of High-Dose Glycine Therapy

There is substantial third-party objective evidence that O experienced unrelenting illness that generally increased in the 10 years that preceded glycine treatment and that O's education and social life were profoundly disrupted. 

Evidence for disruption of education resides in a comprehensive set of third-party records on school attendance and nonattendance, academic performance during attendance, results from standardized academic tests, financial records from academic institutions, and written correspondence from academic administrators and city truancy officials. For 8 years before glycine treatment, there was no attendance of school or commercial tutoring. Resumption of education following the initiation of glycine treatment is likewise supported by comprehensive third-party objective evidence. Over a period of approximately 2 years, evidence for sustained and increasing improvement resides in the fact that O passed the high school equivalency exam, prepared for the SAT test in a commercial course, preformed well on SAT test and began college. Academic records indicate excellent attendance and performance. 

 Disruption of education was accompanied by disruption of social life and a housebound state. Evidence for a resumption of social life following glycine treatment is likewise supported by substantial third-party objective evidence. For example, evidence for a resumption of social life resides in email correspondence with classmates that relates to social activities at specific events and locations in his city and another city. Credit card records indicate the purchase of clothes, attendance at restaurants and concerts in his city and another city. Thus, there is clear, third-party objective evidence for resumption of an active social life and normal mobility in his city that is not subject to concerns about subject, parental, or researcher bias relating to outcome assessment. This evidence for a major reduction in preglycine life disruptions suggests a major improvement in the OCD and BDD dimensions of O's illness. However, before this major improvement can be considered a genuine effect of glycine, it is necessary to rule out placebo effects and a spontaneous remission. 

 Given that placebo effects derive from patient anticipations for treatment, there is the a priori expectation that placebo effects will not be durable in individuals experiencing powerful, life-disruptive psychiatric illness. This expectation has been confirmed by Quitkin and coworkers in systematic studies of placebo effects in depression [57–61]. Specifically, there is a marked tendency for placebo responses to occur abruptly within the first two weeks and to disappear within the first 12 weeks of treatment. This placebo response pattern is in contrast to what the authors regard as the response pattern for a true pharmacologic response, where the initial effects appear after a period of two weeks and tend to last 12 weeks or longer. The placebo response pattern can, of course, be seen either with a true placebo or with an active drug. These authors “conclude that a significant portion of relapses within the first 6 weeks of treatment with an active drug are not related to loss of a true drug effect. Rather some are related to loss of nonspecific placebo effects, and abrupt nonpersistent responses during drug treatment are most likely the result of placebo effects” [60]. They note that the early occurrence of placebo effects is consistent with the notion that they derive from patient expectations [61]. Others have made similar observations [62]. In relation to studies of OCD patients, Ackerman and Greenland [63] have noted that the observation of less improvement in longer trials of active drugs may be due to placebo effects from expectations generated by side effects: “It is possible that in the early 10-week clomipramine and 8-week fluvoxamine trials, some of the subjects in the active treatment arms responded to nonspecific study effects (such as side effects). In longer studies, that improvement was not maintained.” Here it can be noted that Quitkin et al. [59] suggest that “precise assessment of drug effects probably requires several months of observation.” 

 In view of the above considerations, we suggest that it is highly unlikely that the objective, major, and long-lasting changes in life activities that followed glycine treatment represent a placebo effect. Unlike placebo effects, the change that followed initiation of glycine treatment was not abrupt. The subject and his parents report that the first sign of improvement (leaving home without parental escort for the first time in 5 years) occurred 5 weeks after initiation of glycine consumption. Further improvement was also gradual, with third-party objective evidence documenting a gradual resumption of education and social life over treatment periods totaling 150 weeks (in an observation period of 5.3 years). This is much longer than the “several months” described by Quitkin et al. [59] as sufficient to detect true drug effects. 

 Although the above studies clearly suggest that effects generated by patient expectations tend to be transient, it is nonetheless possible to observe some cases in which improvement from a known placebo appears to be enduring. For example, Quitkin observed sustained improvement in placebo-treated individuals with depression [57]. In these cases, the authors make the reasonable suggestion that the improvement was more likely to have been a spontaneous remission than a placebo effect. Therefore, it is necessary to consider the possibility that O's enduring improvement following initiation of glycine treatment was due to spontaneous evolution of illness. 

 We first note that a spontaneous remission appears unlikely on the basis of prior longitudinal studies, which suggest that both OCD and BDD generally have a continuous course, with spontaneous remissions being rare, especially in SSRI-refractory cases [64–69]. Moreover, the occurrence of both disorders together tends to be associated with a worse prognosis. Although suggestive, these considerations do not constitute definitive evidence against a spontaneous remission in a specific case. 

 One approach to distinguish true efficacy from spontaneous remission in a specific case is to determine if deteriorations occur during periods of nontreatment. To pursue this approach, this study was continued until we obtained objective, third-party evidence for an unequivocal deterioration during nontreatment that avoided concerns about subject expectations and outcome assessment bias in the observations made by the subject, his parents and researchers. 

 As noted for the long off-glycine periods, OP6 and OP10, modest relapses were seen for OCD and BDD. However, the clearest evidence of deterioration was for the cognitive dimension of O's illness. Most notably, third-party objective evidence derived from clinical neuropsychological testing in OP10 suggests substantial deficits in selective aspects of cognition by the end of this period. This testing with formal methods was done by a neuropsychologist who was blinded to psychiatric and treatment histories. A comparison of this off-glycine data with the excellent SAT scores obtained in the on-glycine period, OP5, suggests a substantial decline in selective aspects of cognition in OP10 relative to OP5 levels, as discussed in Section 3.5.11. All of this evidence is objective except for some neuropsychological tests that required subjective evaluation by the clinical neuropsychologist, such as the Rey-Osterrieth test. In these cases, any subjective judgments were made under blinded conditions that precluded expectations of the neuropsychologist that were based on treatment history or psychiatric diagnoses. 

 The possibility that treatment cessation could have generated expectations in our subject that somehow affected his performance on the neuropsychological testing can also be considered. We first note that the subject reports that he initiated testing because of speech difficulties. The evaluation revealed normal verbal fluency but did reveal multiple other deficits. The point here is that the deficits found were different from the subject's initially expressed complaints. Further evidence against expectations for a decline in memory after glycine cessation reside in the fact that in serial interviews, the subject has expressed doubts about the validity of the tests for memory deficits and has noted that friends have commented on his excellent memory of the distant past. Moreover, he has not resumed high-dose glycine consumption in response to the test results. It can also be noted that the neuropsychologist reported that the subject was euthymic and appeared to make a full effort. 

 On the basis of the above considerations, we suggest that the third-party evidence that we present for a cognitive deterioration is either fully objective evidence or is evidence that is not compromised by an absence of blinds or by subject expectations. An important point is that the selective cognitive deterioration was substantial, since some WMS-III scores were in the first percentile and since the Rey-Osterrieth copy score was more than 8 standard deviations below the mean. We suggest that it is unlikely that scores on these tests would have been much lower had they been measured before the initiation of glycine treatment. 

 In summary, we suggest that the bias-free evidence that we present for a major improvement of OCD and BDD during treatment and for a major deterioration of selective aspects of cognition during nontreatment is consistent with the possible efficacy of glycine.

### 4.2. The Findings of This Case Study May Have Generality

In response to the success achieved in the first year of this study, a collaborative initiative was begun to test glycine with other OCD patients. The first aspect of this initiative has been a placebo-controlled study in which glycine was used as an adjunct to pre-existing pharmacotherapy. The 5 individuals who were treated with glycine had a mean decrease in Y-BOCS [70] score of 6.04 points where as the 9 subjects treated with placebo experienced a 1.00 point decrease (*P* = .053) (Greenberg, Benedict, Doerfer, Perrin, Panek, Cleveland, and Javitt, Adjunctive glycine in the treatment of obsessive-compulsive disorder in adults [71, 72]). Although much larger studies are needed to determine the frequency of responders, the results of Greenberg et al. are in agreement with this case study and raise the expectation that additional OCD patients will be glycine responders. To facilitate large trials, it would be desirable to identify glycine-responsive subtypes. The comprehensive case description in this paper has been motivated, in part, by this goal.

### 4.3. The Results of This Case Study Have Been Achieved with a Single Psychotropic Reagent

To our knowledge, this case study represents the first use of high-dose glycine with OCD or BDD and the first use of glycine alone with any psychiatric disorder. In addition, there was no psychotherapy or behavior therapy during the 5-year period of monitoring. Thus, this study is free of factors that confound the interpretations of many studies. Given the magnitude of the improvement seen in this study, we suggest that trials of glycine alone be considered for future studies.

### 4.4. The Results of This Case Study Motivate Long Trials of Glycine

In agreement with prior studies of glycine as an adjunct to conventional medication in schizophrenia [73], an initial response to glycine treatment was detected in this study only 23 days after reaching the target dose. However, the improvement reported for the first 12 weeks of treatment was only a small fraction of the improvement reported for the five on-glycine treatment periods that extended over a period of almost four years. This suggests that the short clinical trials used in the past, for example, 12 weeks, may have failed to reveal the full therapeutic potential of high-dose glycine treatment in the disorders studied.

### 4.5. Do the Results of This Case Study Support the Hypo-NMDAR-ST Hypothesis?

The results of this high-dose glycine trial with refractory OCD, BDD, and cognition deficits are in unequivocal agreement with the main prediction of the Hypo-NMDAR-ST hypothesis that inspired its initiation. However, it is also necessary that this hypothesis be compatible with prior, well-established findings for NMDAR neurotransmission. 

 Since our hypothesis was constructed to explain the apparent behavioral effects of clarithromycin, which has been reported to inhibit NMDAR-ST at a downstream point, it is reasonable to consider if other downstream NMDAR-ST inhibitors have similar effects. There are many studies of NMDAR inhibition on human behavior, but, to our knowledge, all employ global inhibitors, such as phencyclidine and ketamine, that act on the NMDAR itself. Thus, a direct comparison is not possible. With this caveat in mind, it is nonetheless of interest to consider known effects of ketamine and phencyclidine in relation to this study. 

 To our knowledge, controlled studies with phencyclidine and ketamine have considered either healthy subjects or subjects with schizophrenia [38, 74]. In healthy subjects, the basic findings are deficits of cognition and other signs and symptoms that are schizophrenia-like [38, 74]. In subjects with schizophrenia, a worsening of existing or reactivation of previously controlled symptoms is the main presentation [38, 74]. In these very short-term experiments, signs and symptoms of OCD, to our knowledge, have not been reported. Although visual distortions of body parts are a prominent consequence of ketamine and phencyclidine [38, 74] and somatosensory abnormalities have been suggested to be an aspect of BDD deriving from parietal-occipital abnormalities [75, 76], full-fledged DSM-IV BDD has not, to our knowledge, been reported in these experiments. At first sight, these findings might be considered incompatible with our hypothesis. However, general considerations suggest that local defects in NMDAR-ST could be specific for OCD/BDD. The absence of such defects in normal individuals and in individuals with schizophrenia would explain the absence of OCD/BDD in the reported ketamine and phencyclidine experiments. Here it can be noted that disorder-specific defects appear to have influenced the exacerbations induced by clarithromycin in other cases. For example, in one case, previously controlled bipolar disorder was reactivated [77]. In another, previously controlled posttraumatic stress disorder was reactivated [78]. 

 It can also be noted that disorder-specific defects for OCD and BDD are plausible in relation to known neurobiology. Given that SSRIs are the first line drugs for both OCD and BDD, it is possible that abnormal serotonin neurotransmission plays a causal role in these disorders in some individuals. This raises the possibility of a local, OCD/BDD-specific defect that is restricted to serotonergic neurons of the raphe nuclei. Recent evidence indicates that NMDAR-ST regulates the firing rate of these neurons [79], suggesting that deficient NMDAR-ST could lead to an abnormal firing rate. The more downstream the defect the more likely it will involve the molecular systems that produce, release, and transport serotonin. Recent clarifications of mechanisms regulating serotonergic neurotransmission [80–84] reveal a complex picture in which multiple processes couple NMDAR input with serotonin release/reuptake. Defects occurring in these processes could represent OCD/BDD-specific defects in NMDAR-ST. 

 Another possibility is a deficiency of NMDAR-ST in GABA-ergic inhibitory interneurons (abundant in both the dorsal and median raphe nuclei [85]) that regulate serotonergic neurons. This scenario can be seen as a variant of the elegant and influential theory of Olney et al. in which deficient NMDAR-ST leads to loss of feedback inhibition [37]. The core principle of this theory, which goes beyond “hyperglutamatergic” or “hypoglutamatergic” models [14], is that glutamate “functions not only as a straightforward excitatory agent in the brain, but as a major regulator of inhibitory tone” [37]. Local NMDAR-ST deficits restricted to interneurons that regulate serotonergic neurons could reasonably be expected to lead to abnormal serotonergic tone and the emergence of OCD/BDD. 

 In both of the above scenarios, there is an absence of the more widespread perturbations expected for global NMDAR-ST inhibition by ketamine and phencyclidine. The absence of these perturbations would be compatible with the emergence of OCD/BDD without the schizophrenia-like symptoms seen with ketamine and phencyclidine. Also, in both scenarios, it would be reasonable to expect worsening of signs and symptoms by an inhibitor and improvement from an enhancer of NMDAR-ST. However, it should be emphasized that the above analysis is intended only to suggest plausibility of disorder-specific defects, not to propose a specific mechanism for the case under study. 

 Although OCD and BDD have not been seen in ketamine experiments with humans, some of the cognitive deficits seen in O show substantial overlap with the cognitive effects of ketamine. A consistent finding in multiple ketamine studies is a robust, dose-dependent decrease in verbal declarative memory that occurs at doses below those that induce schizophrenia-like symptoms [38, 86]. Deficits in verbal memory and learning in ketamine experiments have been assessed with story recall and list learning tests. For example, Newcomer et al. [86] found a dose-dependent decrease in verbal memory under ketamine using a paragraph recall test from the Wechsler Memory Scale-Revised [87]. Immediate and delayed recall scores from this study can be compared with O's corresponding scores on a revised version of this test, the WMS-III. O's Auditory Immediate and Auditory Delayed scores were in the ~1st percentile. In other ketamine studies [88–90], a robust decline in verbal memory has been found with list learning tests such as the Hopkins Verbal Learning Test (HVLT) [91]. These results can be compared with the significant deficits seen in O's scores on the CVLT-II [48], where the T score for Trials 1–5 was ~36 (*Z* = − 1.4) and Z-scores for delayed recalls (free and cued) ranged from ~−2 to ~−2.5, except for the short delay free recall, which was ~−1. The Z-score for total hits was ~−4. Thus, O's off-glycine deficits in verbal memory are strongly reminiscent of those induced by ketamine. This similarity is clearly supportive of our hypothesis. Also supportive are O's very high on-glycine scores on the GED and verbal SAT tests (99th and 90th percentiles, resp.), which suggest much improved verbal learning and memory in an on-glycine state, as discussed above. 

 The finding that ketamine-induced cognition deficits appear at doses lower than those needed for other types of symptoms [38, 86, 92–94] is also of relevance to our hypothesis. Assuming that an endogenous NMDAR-ST defect would mimic ketamine, this finding leads us to expect that the return to a deficient NMDAR-ST state after cessation of glycine would manifest itself with a cognitive relapse whose emergence is earlier and more pronounced than the relapses for other categories of signs and symptoms. Our data from OPs 6 and 10 appear to be consistent with this prediction. Also of relevance is the fact that in the initial emergence of illness in early childhood, difficulties in cognition appeared before obsessive-compulsive behaviors and BDD-related signs and symptoms. 

 Finally, we note that additional support for our Hypo-NMDAR-ST hypothesis can be found in the course of O's illness. Specifically, it is known that NMDAR inhibition has mild behavioral effects in childhood and much larger effects after puberty [95–100]. Assuming that an endogenous NMDAR-ST defect would imitate an inhibitor, it follows that O's very mild illness in childhood and the emergence of much more incapacitating illness after age 15 are consistent with an NMDAR-ST deficit.

### 4.6. Do the Results of This Case Study Support Alternative Hypotheses?

Although the findings of this study support the Hypo-NMDAR-ST hypothesis on which it is based, it is appropriate to consider our findings in the light of alternative hypotheses proposed by others. 

#### 4.6.1. Is O's Case an Example of PANDAS?

One of the defining features of the PANDAS subtype is the occurrence of exacerbations with sudden, abrupt onset [25]. The exacerbations are described as “typically quite dramatic, with patients reporting that their symptoms “…came on overnight” or “…appeared all of a sudden a few days after I had a sore throat.”” The fine details of O's exacerbations are not congruent with this picture. For O's age-19 exacerbation that followed a positive throat culture, detailed notes of O's father indicate an improved relationship with his son during the acute phase of the infection. The behavioral deterioration is described as beginning “as soon as the infection cleared.” Worsening of existing symptoms (e.g., line-crossing obsession) occurred 24 days after the positive throat culture. Episodes of violent imagery appeared for the first time approximately 40 days after the positive throat culture. In addition to the slower kinetics, O's nonresponse to IVIG therapy is unlike some PANDAS patients [26].

#### 4.6.2. Are the Findings for O's Case Compatible with an Autoimmune Etiology?

Although, O's case cannot be classified as a clear case of PANDAS, it is still possible that it involves an antigen-specific autoimmune response induced by GABHS. There are, of course, multiple precedents, both experimental and theoretical, for the induction of antigen-specific anti-self-immune responses by foreign antigens that cross-react with self-epitopes or interact with self-receptors [101–103]. Thus, the occurrence in O of two exacerbations following probable GABHS infections is immediately suggestive of an antigen-specific autoimmune mechanism. This follows from the major precedent residing in rheumatic fever (RF) as an antigen-specific autoimmune disease primed by GABHS. A comparison of O's illness with RF is therefore warranted. 

 As expected for a pathogen-induced, antigen-specific autoimmune disease, recurrences of RF in adulthood are associated with probable reinfection with GABHS, often as a result of known, close contact with children having GABHS infections [104]. The time interval between an initial occurrence of acute RF in childhood and a subsequent occurrence in adulthood can be multiple decades. During these long periods, there is an annual occurrence of upper respiratory viral infections, some of which are “flu-like.” As expected for infections which are unlikely to be antigenically related to GABHS, there is no retriggering of RF. Another point is that penicillin prophylaxis is effective in preventing a return of RF [105]. This would not be expected if viral infections significantly trigger recurrence of RF. 

 In contrast to RF, exacerbations in O's case and in some PANDAS cases [25] appear to follow both GABHS and non-GABHS infections. It appears that Sydenham's chorea, a sequela of RF, can reoccur without GABH infection [106]. One might propose that, in these cases, antigen-specific clones primed by an initial GABHS infection are somehow retriggered by other pathogens, such as viruses. However, the failure to see this phenomenon in RF represents a major challenge to this proposal. 

 One possibility is that the putative brain injury responsible for O's case arises from nonantigen-specific consequences of GABHS and other infections. Such a mechanism would be compatible with recurrence from both GABHS and non-GABHS infections and would not be incompatible with the peripheral antibrain antibodies reported in the recent literature [107–111], since the latter could be a result (rather than a primary cause) of brain injury due to the release of antigens from an immunologically protected site. Here it is of interest that Husby et al. who in 1976 found antistriatal antibodies in patients with Sydenham's chorea [112], also found similar titers of antistriatal antibodies (using identical techniques) in patients with Huntington's chorea [113]. This led in 1977 to the suggestion that Huntington's chorea could be an infection-triggered autoimmune disease. The latter is now known to be a polyglutamine disease [114] that involves preferential death of striatal neurons [115]. In brain-injury models of this type, it is formally possible that antibrain antibodies arising from brain injury could play a secondary role in pathogenesis. However, the demonstration that such antibodies play a causal role is a difficult task that is technically inaccessible in the absence of validated animal models [116, 117], which are unavailable for psychiatric disorders that involve high-level human thinking.

#### 4.6.3. Is O's Illness due to a Primary Vascular Defect?

**Figure 1:**
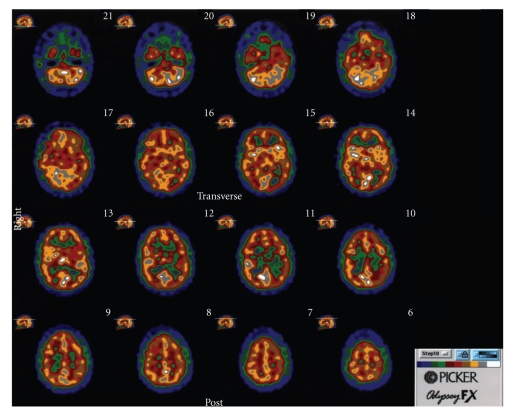
Age-22 SPECT scan showing prominent and heterogeneous hypoperfusion. One color step equals a 10% change in perfusion. The highest perfusion is indicated by white. See text for acquisition conditions and Table 4 for results of semiquantitative analysis.

The four SPECT scans done over five years provide evidence for stable and sustained hypoperfusion (Figure 1, Appendix A.3). The cause of this hypoperfusion is unknown. One possibility is that it reflects a primary vascular defect of long standing that creates a susceptibility to psychiatric illness. In this context, one is reminded of the interesting proposal by Hanson and Gottesman that vasculature-inflammatory defects are a root cause of psychiatric illness in subsets of patients with schizophrenia and other psychiatric illnesses, including those associated with GABHS infections [118]. They postulate “a chronic smoldering inflammation of the vasculature” that is episodically triggered to cause brain injuries leading to psychiatric illness [118]. O's abnormal SPECT scans (with a differential diagnosis that includes “vasculitis”) and his exacerbations following infections suggest consideration of the theory of Hanson and Gottesman in future studies.

### 4.7. Are O's SPECT Scans Typical of Other Patients with Similar Diagnoses?

In the literature on OCD, SPECT scans most commonly show hyperperfusion in frontostriatal areas, but hypoperfusion is prominent in a significant fraction of patients [119, 120]. To our knowledge, in cases where hypoperfusion was seen, heterogeneity was not noted. In a rare SPECT study of BDD, Carey et al. reported that both regional hypoperfusion and hyperperfusion could be seen in six subjects with BDD [75]. All showed hypoperfusion in the temporal region. Of special interest are 2 young adult male BDD patients with no other current comorbidity who showed heterogeneous hypoperfusion in the occipital and parietal lobes. As seen in Figure 1 (Appendix A.3), heterogeneous hypoperfusion is a distinctive feature of O's SPECT scans, suggesting that his scans most closely resemble the BDD scans.

### 4.8. Possibilities for Improved Monitoring and Treatment

**Figure 2:**
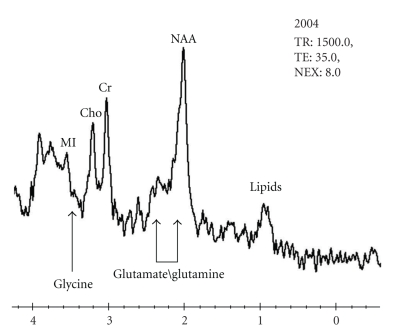
Magnetic Resonance Spectrum from 2.5 cm Voxel in Right Medial Frontal Region 4.5 Hours after Consumption of 25 Grams of Glycine + 3 Grams of Arginine. TE: echo time; TR: repetition time; NEX: no. of excitations; MI: myo-inositol; Cho: choline; Cr: creatine; NAA: N-acetylaspartic acid. See text for additional details.

The detection of brain glycine by single voxel MRS was initially accomplished only in individuals with genetic or other defects that lead to unusually high levels of glycine [121, 122]. Our preliminary results in 2004 with a 3 Tesla machine (Figure 2, Appendix A.4) raise the possibility that high-dose glycine treatment causes sufficient elevation of forebrain glycine to permit detection using standard procedures with optimal echo times. Future attempts to confirm this finding will benefit from recent improvements in techniques and the emergence of 7 Tesla scanners [123–125]. 

 The opportunity to monitor glycine concentrations in specific brain regions will permit the exploratory studies needed to identify the oral dose required to get the desired concentrations in relevant brain regions. Studies in rodents suggest that in much of the forebrain, endogenous glycine concentration is substantially lower than that in the hindbrain and spinal cord [126], making the ability of MRS to measure glycine concentrations in small voxels an important advantage. As noted previously, high-dose glycine treatment may lead in some individuals to mildly elevated plasma ammonia. This could lead to elevated levels of ammonia in the brain. Studies in rats have indicated that moderate levels of chronic hyperammonemia inhibit NMDAR neurotransmission [127]. This raises the possibility that optimal glycine therapy in individuals with elevated ammonia would require a coadministered agent to control ammonia. There is also the possibility that excess levels of glycine in the brain may lead to increased in situ production of ammonia. These issues can likely be addressed with the new high-field scanners [128–130].

## 5. Conclusion

Trials of glycine with schizophrenia have prompted a search by pharmaceutical companies for drugs that enhance NMDAR neurotransmission. While this study suggests that these drugs may also be useful with OCD and BDD, it is likely that a significant period of time may elapse before they become available. We therefore emphasize that glycine is available here and now and at very low cost. This point is of great significance to patients, who need immediate relief from what is often severe and unrelenting illness. With these considerations in mind, we suggest that the findings of this study motivate further exploration of the efficacy and safety of glycine as a treatment of OCD and BDD as well as further study of the Hypo-NMDAR-ST hypothesis that motivated this treatment.

## Figures and Tables

**Table 1:** Glycine consumption in the ten observation periods*.

Observation Period	Start Day	Stop Day	No. Days	Average	%Days	%Days	%Days	%Days	%Days	%Days
				Gly/day gm	50–66 gm	40–49 gm	30–39 gm	20–29 gm	10–19 gm	0 gm
1	1	203	203	44.6	50.7	23.6	1.0	20.2	1.5	3.0
2	204	244	**41**	**0**	0	0	0	0	0	**100**
3	245	371	127	36.0	55.9	0.8	1.6	28.3	0	13.4
4	372	411	**40**	**0**	0	0	0	0	0	**100**
5	412	548	137	33.3	34.3	20.4	5.8	19.7	1.5	18.2
6	549	768	**220**	**0**	0	1.4	2.3	3.6	1.4	**91.4**
7	769	1022	254	37.5	50.8	2.8	3.1	27.2	1.6	14.6
8	1023	1068	**46**	**0**	0	0	0	0	0	**100**
9	1069	1400	332	43.1	59.0	4.2	0.6	27.1	2.7	6.3
10	1401	1941	**541**	**0**	0	0	0	0	0	**100**

*Amounts indicated are amounts weighed. Amounts taken are less due to the tendency of glycine to settle and leave a residue in the drinking glass.

**Table 2:** Summary of Changes in OCD and BDD in the ten observation periods.

OP1	On-glycine 203 days	Partial reduction of OCD and BDD. Partial elimination of housebound state. Increased mirror tolerance. Barbershop haircuts. Partial resumption of social life. Attendance at social gatherings at homes of family friends. Increased volition and study effort. Obtained GED diploma. Improved hygiene and attention to clothes. Sleep cycle spontaneously normalized.

OP2	Off-glycine 41 days	Partial loss of OP1 gains following infection with high fever. New hand washing ritual based on fear of harm with mild impairment.

OP3	On-glycine 127 days	Rapid recovery from relapse in OP2 and further improvements in OCD and BDD beyond those of OP1. Attended preparatory course for SAT.

OP4	Off-glycine 40 days	No significant change in OCD or BDD.

OP5	On-glycine 137 days	Further reduction in both OCD and BDD. Resumption of normal family social life in home for first time in 8 years. Preparation for SAT by self-study. Excellent SAT scores greatly increased from initial practice test scores (see section on cognition). Housebound state eliminated but community movement remained partially restricted. Improvements resistant to stress and disappointment. Hand washing ritual that started in OP2 disappeared by end of OP5.

OP6	Off-glycine 220 days	Entered college at beginning of this period. In-class socialization with peers. Good academic performance except for apparent cognitive decline after 21-26 weeks (see section on cognition). No significant change in BDD. Increased somatic/cognitive preoccupations with mild impairment. After 10 weeks, a mild increase in line-crossing obsession.

OP7	On-glycine 254 days	Transferred to more competitive college. Consistent academic effort with respectable grades. Major reduction in OCD/BDD. No sensitivity to crime reports in news and increased community mobility. Resumed hair combing in front of mirrors with partially covered face. Father-related BDD by proxy disappeared. Improved family socialization and more order at home. Somatic/cognitive preoccupations remained mild. Gains resistant to stress.

OP8	Off-glycine 46 days	No significant change in OCD or BDD.

OP9	On-glycine 332 days	Very high grades. Developed career ambition. Largely complete remission of line-crossing obsession. Full community mobility and socialization with peers. First date with a girlfriend in 13 years. Reduced self-related BDD and mother-related BDD by proxy. Reduction of somatic/cognitive preoccupations towards end of this period. Improved orderliness at home.

OP10	Off-glycine 541 days	Increase in self-related BDD and in mother-related BDD by proxy caused difficulties in social interactions with mother, relatives, and family friends. Reduction in community mobility due to partial return of major line-crossing obsession. Return of difficulty in making eye contact during greetings and conversations. Reduced volition and orderliness at home. Partial relapse of OCD and BDD occurred very slowly. Major decline in selective aspects of cognition relative to OP5.

**Table 3:** Summary of cognitive and educational data.

Age 7	Formal cognitive testing suggests mild deficits, including signs of deficits in attention and working memory.

Age 14	Poor retention of algebra topics during intensive paternal tutoring. Same types of math errors seen in Age-7 cognitive testing.

Age 17–25	No regular school or formal tutoring in the eight years preceding glycine treatment.

Age 25	GED test taken in standard time period 145 days after initiation of glycine treatment. Five GED subtest scores ranged from the 67th to the 99th percentile.

Age 27	SAT taken in standard time period 68 days after resuming glycine in OP5. Verbal score = 90th percentile. Math score = 50th percentile. Official scores were each 120–130 points higher than the lowest practice tests.

Age 27	Paternal impression of rapid absorption of new math topics is supported by college placement exam taken just after end of glycine consumption for 138 days. College education initiated. Generally good academic performance in English and Algebra courses. The same types of math errors seen at ages 7 and 14 returned 21–26 weeks after glycine cessation.

Age 30	Formal cognitive testing 17 months after glycine cessation revealed substantial deficits in tasks requiring manipulation of nonsequential items, self-monitoring, response inhibition, and set shifting. Visual-perceptual and visuoconstructive skills were found to be severely impaired. Auditory immediate and delayed scores on subtests of the Wechsler Memory Scale III were in ~1st percentile. Memory deficits were also revealed by the California Verbal Learning Test-II. A difference of 30 points between predicted and actual results was found for the Working Memory Index of the Wechsler Test of Adult Reading (cumulative percentage = 1%), suggesting a cognitive decline from OP5 where high verbal SAT performance was seen.

**Table 4:** Count rates in counts per minute for Age-22 SPECT scan shown in Figure 1.

Slice location	Region	Left	Right
	Basal ganglia	1313 (+1.7%)*	1322 (+2.4%)
	Thalamus	1205 (−6.6%)	1182 (+8.4%)
	Cerebellum	1311 (+1.6%)	1270 (−1.6%)
2.5 cm above canthomeatal line			
	Medial frontal	970 (−24.8%)**	949 (−26.5%)
	Lateral frontal	974 (−24.4%)	974 (−24.4%)
	Anterior temporal	1243 (−3.7%)	1283 (−0.6%)
	Posterior temporal	1189 (−7.9%)	1066 (−17.4%)
5.0 cm above canthomeatal line			
	Occipital	1027 (−20.4%)	1047 (−18.9%)
7.0 cm above canthomeatal line			
	Superior frontal***	1002 (−22.4%)	1059 (−17.9%)
		1007 (−22.0%)	1029 (−20.2%)
	Midparietal***	1059 (−17.9%)	1111 (−13.9%)
		997 (−22.7%)	1102 (−14.6%)

*Percent differences are relative to the average of the cerebellum count rates.

**A reduction >12% is considered clinically significant.

***For superior frontal and midparietal regions, duplicates were quantitated.

## Appendix

### A. Details of Preglycine Illness and Medical/Radiologic History

#### A.1. Signs and Symptoms of Illness in the Preglycine Period

##### A.1.1. OCD Signs and Symptoms

Obsessions In the preglycine period, O and his parents report that obsessive mentation was continuously present from the time of eruption of frank illness at age 15. The most impairing obsession was a line-crossing obsession, which would be triggered (or intensified) when O would see a person in his neighborhood whom he considered to be likely to commit a violent, personal crime. Reports of crime in the news media also activate the obsession, provided that they occur in a “good” neighborhood. Crime events in a “bad” neighborhood are of no concern, since, in the latter case, there is evidently no line-crossing. The line-crossing obsession is highly ego-dystonic. It is also a “reactive obsession” in the classification framework of Lee and Kwon [131], in which obsessions responsive to perceived environmental stimuli are called “reactive.” By not leaving his home O was able to reduce distress by avoiding exposure to environmental intensifiers, but this generated major impairment by disrupting education and social life.

Obsessions involving violent imagery are also an important feature of O's illness. The first occurred during the age-19 exacerbation that followed bilateral ear infections. Violent imagery involved images of a knife cutting his father causing extreme fear and distress. As O's father noted in a letter to a psychiatrist: “The terrifying thoughts are truly agonizing and he begs for relief while holding my hand and literally trembling.” It should be noted that two psychiatrists, who were consulted at this time, concluded that the violent imagery did not involve hallucinations. Violent imagery with intense distress has occurred only during the three major exacerbations and thus represents a distinctive “signature” of such exacerbations. Since these episodes are not triggered by perceived environmental stimuli. O's episodes of violent imagery can be classified as “autogenous obsessions” in the classification framework of Lee and Kwon [131]. It is of interest that O reports that episodes of violent imagery are not associated with anger, which is in contrast to reactive obsessions.

Ritualistic Behaviors According to O's father, activation of the line-crossing obsession would invariably lead to a stereotypic conversation that was highly repetitive due to frequent but incomplete attempts to make a particular point. O's father would provide repeated reassurances that O's obsession-related fears were unfounded. O's father refers to this type of conversation as a “talk-through.” Talk-throughs were not restricted to the line-crossing obsession, but could concern any OCD-related obsession or the BDD-related excessive preoccupations (see below). O's father emphasizes the distinct, “qualitative” difference between a talk-through and a normal conversation that progresses in a linear fashion from one point to another.

Although talk-throughs have features of reassurance rituals, they are more complex as they frequently involved an outburst of verbal anger. O's father notes that when talk-throughs were avoided for several days there would be a stronger outburst of anger when they were resumed. After about 1 hour of the repetitive conversation and after an outburst of anger, the psychic distress, including anger, would usually be dissipated until they returned the next day. Talk-throughs were a daily feature in much of the six year period that preceded high-dose glycine treatment. At times of peak illness, they consumed multiple hours per day.

Other ritualistic behaviors are reported to have included prayer, wall-touching, jumping into bed, and turning lights on and off and were done in response to fear of harm. O indicates that fear of harm could either be for himself or for his parents, especially his father. Most of the rituals done to prevent harm had to be done “just right.” In some cases, rituals had to be done a large number of times or according to a numerical pattern (in groups of 30 in one case).

Prior to the age-22 exacerbation that followed antibiotic treatment for *H. pylori*, there were a number of “just right” rituals but they were minor in relation to the reassurance rituals associated with the line-crossing obsession. Following the antibiotic treatment for *H. pylori*, O's father's diary indicates a very large increase, over a period of about two months, in a prayer ritual in which O would suddenly drop to his knees. Dropping to his knees had to be done “just right.” This ritual was frequently performed throughout an entire day, leading to knee calluses.

##### A.1.2. BDD Signs and Symptoms

In periods when O's BDD signs and symptoms were at their peak, O's father reports that O would spend most of a day “working on the mirror,” an activity that would involve repeatedly checking his appearance in a mirror in order to get a good impression of his face. Rare good impressions were always followed by numerous disturbing impressions that were often associated with verbal anger. O's parents report that this activity clearly appeared to intensify distress and to increase O's preoccupation with his presumed facial defects.

In addition to the above self-related BDD, there was a “BDD by proxy” for his mother's and father's faces. Specifically, there was the fear/belief that they were ugly and that he looked like them. “BDD by proxy” first emerged at age 19, several months after the first infection-associated exacerbation and led to a general avoidance of his mother and restricted viewing of his father.

In periods when O was not spending multiple hours per day “working on the mirror,” there was complete avoidance of mirrors or other reflective surfaces or photographs of himself. Activities were restricted by mirror avoidance and inability to handle photo-identification. Likewise there was avoidance of social events as a result of a fear that someone would say that he was ugly or looked like his mother or father. All of this reinforced O's tendency to be housebound, adding to the similar effects of the line-crossing obsession. Being housebound reduced distress but led to major impairment by disrupting education and social life.

##### A.1.3. Somatic Preoccupations

Somatic complaints associated with dysphagia, muscular discomforts and discomforts at joints and the vasculature (cold hands, hot hands, palpitations) are another distinctive aspect of O's behavior. The sites of discomfort are reported to have been stable over time, although complaints at a particular time usually involve only a subset of sites.

The possibility that O's somatic complaints represent hypochondriasis was reviewed by the OCD specialist consulted at age 19. O's father's notes and the chart for these consults indicate that hypochondriasis was not found. Thus, O's somatic concerns are not presented as improperly held beliefs or fears about specific illnesses; rather, they are presented as complaints about discomforts at body sites with claims that they impede his functioning. Thus, O's somatic complaints are different from the somatic obsessions often reported for OCD, which are typically concerned with fears of illness rather than discomforts at body sites [132].

In the recent interviews, O has reported that discomfort in a particular joint increases gradually until it reaches the point where he feels a need to “pop” the joint to eliminate the discomfort. The discomfort gradually returns and the cycle repeats. It appears that the action of joint popping does not in itself generate impairment, since it is done only a few times per day. Here we note that O never received a diagnosis of a tic disorder. Moreover, it has not been possible to identify any tic-like behaviors for O's other somatic preoccupations.

One possible explanation of O's somatic preoccupations is that they reflect, at least in part, an intrinsic hypersensitivity to body sensations. This has been described for individuals with Tourette syndrome, which, historically, has been known to occur often with OCD [133] and which may therefore share causal factors. The joint discomfort and its relief by “popping” closely resemble the site sensitivity and premonitory urges reported for individuals with TS [134]. As Cohen and Leckman have noted [135]: “Patients with TS are remarkably sensitive to and are easily captured by changes in the sensory world…” Although, O has not received a diagnosis of TS and does not appear to cross the threshold for a tic disorder, it is possible that TS and OCD as it occurs in O share causal factors, one or more of which generate the sensory hypersensitivity. This possibility is supported by a more recent characterization of sensory phenomena in OCD, OCD+TS, and TS [136].

It should be emphasized that in the preglycine period, somatic preoccupations were seen by O's parents to be “almost negligible” in relation to major OCD and BDD presentations. It was during OP6 (the first lengthy off-glycine period) that these preoccupations were first seen to cause a mild but clear impairment.

##### A.1.4. Cognitive Preoccupations

In addition to somatic complaints, O has frequently complained since age 20 about difficulties in speech production, specifically an inability to find words and physical difficulties in speech production. At the end of Observation Period 10 (see Section 3.5.11), O initiated an examination by a speech pathologist, which revealed no speech deficits but did reveal a memory deficit. In a subsequent neuropsychological evaluation, oral expression was in the “superior” range and represented the highest of all scores. On this basis, we tentatively refer to O's speech complaints as “cognitive preoccupations.” However, it is possible that the speech-related complaints are an inaccurate description of genuine cognitive deficits that were revealed in the neuropsychological evaluation (*see* Section 3.5.11). In our view, available data do not allow a firm conclusion.

*Additional documentary material on methods and behavioral phenomena is at www.informaticpsychiatry.org/glycine*.

#### A.2. General Medical History

Except for his psychiatric disorder, O has been in good general health all of his life. During pregnancy, ultrasound-guided amniocentesis was done to rule out Down syndrome. No chromosomal abnormalities were found. Records indicate that delivery was by C-section because of placenta previa. Apgar scores were 9, 9, and 9 at 1, 5, and 10 minutes, respectively. Birth weight was 7.75 pounds. Maternal and paternal ages at birth were 39 and 36, respectively. Developmental abnormalities were not noted by parents or pediatrician. Infectious disease history has been unremarkable except in relation to exacerbations of psychiatric illness. Raynaud's disease was first noticed at age 21 just after the end of a taper of paroxetine from 70 mg/day to 0 mg/day. It has been witnessed on several occasions in clinical exams and continues to the present day. At age 26, orthopedic and genetic consultations both revealed a mild connective tissue disorder (megacephaly, arched palate, striae distensae, lax joints, and pectus excavatum) that could not be identified with known disorders. Collagen testing revealed no abnormalities [137]. Three echocardiograms, done at ages 21, 25, and 29, were described as normal except for a “trivial aortal insufficiency” that was noted for the first two but not the last, which was done four years after initiation of glycine treatment. Mild, sporadic dysphagia was first noted at age 17. An upper endoscopy at age 29 revealed a Shatzky ring and eosinophilic esophagitis. Other mild conditions are recurrent otitis externa and recurrent oral stomatitis that was noted as early as age 7, when parents recall a prescription for lidocaine.

In general, neurological exams have been unremarkable except for the confirmation of psychiatric diagnoses. However, at age 15, an initial diagnosis of temporal lobe epilepsy was discounted after discovering an electrode artifact in the EEG. Trigeminal neuralgia, a diagnosis that was described as unusual for a 15-year old, was suggested as a possible alternative. The report emphasized that the symptoms could not be generated in the examination. Subsequent recurrences of these symptoms, if any, appear to have been very minimal. Also, at this time, a neuroopthalmologist found a convergence insufficiency that gradually normalized without treatment, as found in a later examination at age 18 by the same neuroopthalmologist. An EEG at age 26 and after 191 days of glycine treatment was normal.

#### A.3. Brain SPECT Scans

Objective evidence for a CNS abnormality resides in four SPECT Scans (technetium-99m-hexamethyl propylamine oxime (Tc-99m-HMPAO)) done at ages 22, 23, 25, and 27. All scans were done under standard clinical conditions for a resting scan. Approximately 20 mCi of Tc-99m-HMPAO were injected into the patient while eyes were open in a quiet, dimly lighted room. No caffeine or medications were taken in the 3 days before the scan. O is a nonsmoker and is right-handed. Approximately one hour after tracer injection, scans were done with a three-headed camera. Images were reconstructed in the transaxial, coronal, and sagital planes. According to the report for the age-22 scan, marked heterogeneous and decreased cortical uptake was noted throughout both cerebral hemispheres. In addition, severe white matter hypoperfusion was also noted. The report also indicates that the observed pattern is often secondary to vasculitis or encephalopathy of an infectious or toxic nature, for example, shearing injuries, cocaine abuse or Lyme disease. O was tested three times for Lyme disease with negative results and is reported to have never experienced a shearing injury. Careful inquiry suggests that he has never engaged in substance misuse. Systemic lupus erythematosus (SLE) is another illness that can be associated with heterogeneous brain SPECT scans. An evaluation by a senior rheumatologist did not find SLE. A representative scan is shown in Figure 1.

A semiquantitative analysis of the age-22 SPECT scan using the procedure of Mountz et al. [138] is shown in Table 4. Values that are 12% or more below the cerebellum average are considered pathological. The right medial frontal region showed the lowest level of perfusion, which was 26.5% below the average of the cerebellar values. As indicated, the frontal, parietal, right posterior temporal and occipital areas are down more than 12%, whereas the basal ganglia, thalamus, anterior temporal, and left posterior temporal are not.

A second scan was done at age 23 and a third at age 25 just before the initiation of high-dose glycine treatment. The fourth scan was done three days after stopping a 138-day glycine treatment period. At the level of clinical evaluation, these three scans are equivalent to each other and to the prior scan. In summary, the SPECT scans show substantial abnormality of unknown cause that is stable over a five year period. No effect from glycine treatment is detectable. However, it should be noted that 3 days after stopping glycine, the brain glycine level may have returned to normal. Further study is required to determine if elevated brain glycine affects regional cerebral blood flow.

#### A.4. Brain Magnetic Resonance Imaging and Magnetic Resonance Spectroscopy

Magnetic resonance imaging (MRI) studies were obtained at ages 15, 20, 25, and 27 and proton spectroscopy (MRS) studies were obtained at ages 25 and 27. The MRI studies at ages 15, 20, 25, and 27 showed an ovoid focus of increased proton density and T2 signal in the right periatrial white matter. The long axis of this lesion is perpendicular to the ventricle and measures less than 1 centimeter.

In the age-25 scan, the T2 hyperintensities were studied more carefully. MR evaluation was performed with and without contrast using the following sequences: T1 sagittal, T1, T2, proton density, FLAIR and diffusion weighted axial images. Following contrast administration, sagittal, coronal, and postcontrast magnetization transfer axial sequences were also obtained. Precontrast magnetization transfer axial sequence was also performed as a control for contrast enhancement on the postcontrast magnetization transfer axial sequences. No abnormal contrast enhancement was observed. The focus of high T2 signal was found to suppress on flair sequence and showed no signs of marginal gliosis to suggest prior ischemia, inflammation, demyelination or infection. Similar smaller lesions with increased T2 signal are seen in the left centrum semiovale. Single voxel magnetic resonance spectroscopy (MRS) was done on the ovoid lesion and on a corresponding region on the left side. Values of N-acetylaspartic acid, choline, and creatine were within normal limits on both sides. Taking all of the evidence together, it is most likely that the T2 hyperintensities are prominent Virchow-Robin spaces. Significant changes are not seen in the 12 year period bracketed by the scans.

The age-25 MRI/MRS study demonstrated normal blood volume throughout both cerebral hemispheres and the posterior fossa. On the diffusion-weighted sequence, no evidence of acute infarct was seen. Other brain scans include a transcranial Doppler sonogram at age 25 (Day 85-OP1(85)), which was unremarkable. A magnetic resonance angiography scan with and without contrast was done at age 26 (Day 19-OP2(222). A normal scan was seen.

At age 27, an MRI/MRS study was done during a period of high-dose glycine consumption (Day 50-OP7(818)). On this occasion, 25 grams of glycine supplemented with 3 grams of arginine were taken over a period of one hour [139]. Three hours after consumption of glycine + arginine, blood was drawn for measurement of plasma glycine (~1350 *μ*M) and plasma ammonia (~30 *μ*M). Approximately 1.25 hours later, the proton magnetic resonance spectrum (3 Tesla, PRESS (point-resolved echo single-shot techninque), TE (echo time) = 35 msec) of Figure 2 was obtained. The voxel was located in the right medial frontal region, the region of greatest hypoperfusion on the SPECT scan of Figure 1.

Of special interest is a “shoulder” of signal at 3.55 ppm that is adjacent to the major myo-inositol peak at 3.61 ppm. This shoulder is expected to reflect contributions from the glycine resonance at 3.55 ppm and the minor myo-inositol resonance at 3.52 ppm. In prior studies with 1.5–2.0 Tesla scanners and short echo times, a clearly detectable 3.55 ppm shoulder has been seen only in conditions where there is a known excess of glycine (e.g., nonketotic hyperglycinemia) [121, 122]. More recent studies suggest that this is also the case with 3 Tesla scanners. For example, in an MRS study of grey matter in a healthy individual (3 Tesla, TE = 40 msec), no shoulder at 3.55 ppm was seen [140]. Similarly, in a study of the medial frontal cortex in an individual with OCD (3 Tesla, TE = 30 msec), no shoulder at 3.55 ppm was seen [141]. The absence of 3.55 ppm shoulders in these spectra of individuals not taking glycine and the presence of such a shoulder in O's spectrum (3 Tesla, TE = 35 msec) are consistent with an elevated glycine level arising from the elevated plasma glycine that followed glycine consumption 4.25 hours before the scan. However, definitive detection of elevated glycine will require on-glycine and off-glycine measurements, and monitoring of the expected metabolism of elevated levels to baseline levels.

O's 3 Tesla spectrum is of also of interest in relation to the prominent GLX peak at 2.02–2.5 ppm. An enlarged GLX peak is consistent with an increased contribution from the glutamine resonance at 2.45 ppm as a result of ammonia production from glycine metabolism in the brain. Note that plasma ammonia is unlikely to be an issue in this instance, since its measured value ~1.25 hours before MRS was well within the normal reference range. A peak at 0.9–1.2 ppm is also seen and is likely to be of lipid origin.

### Conflict of Interest Statement

Drs. Cleveland, DeLaPaz, and Fawwaz are contributors to a patent application filed by Columbia University that is based on this study. Drs. Cleveland, DeLaPaz and Fawwaz declare no other conflicts of interest. Dr. Challop declares no conflicts of interest.
